# Flavonolignan 2,3-dehydrosilydianin activates Nrf2 and upregulates NAD(P)H:quinone oxidoreductase 1 in Hepa1c1c7 cells

**DOI:** 10.1016/j.fitote.2017.04.012

**Published:** 2017-06

**Authors:** Lenka Roubalová, Albena T. Dinkova-Kostova, David Biedermann, Vladimír Křen, Jitka Ulrichová, Jiří Vrba

**Affiliations:** a Department of Medical Chemistry and Biochemistry, Faculty of Medicine and Dentistry, Palacký University, Hněvotínská 3, Olomouc 77515, Czech Republic; b Institute of Molecular and Translational Medicine, Faculty of Medicine and Dentistry, Palacký University, Hněvotínská 3, Olomouc 77515, Czech Republic; c Jacqui Wood Cancer Centre, Division of Cancer Research, School of Medicine, University of Dundee, Dundee DD1 9SY, Scotland, UK; d Institute of Microbiology, Laboratory of Biotransformation, Czech Academy of Sciences, Vídeňská 1083, Prague 14220, Czech Republic

**Keywords:** ARE, antioxidant response element, DHSB, 2,3-dehydrosilybin, DHSC, 2,3-dehydrosilychristin, DHSD, 2,3-dehydrosilydianin, DMSO, dimethyl sulfoxide, Gapdh, glyceraldehyde-3-phosphate dehydrogenase, GCLC, glutamate-cysteine ligase catalytic subunit, GCLM, glutamate-cysteine ligase modifier subunit, HMOX1, heme oxygenase-1, Keap1, Kelch-like ECH-associated protein 1, MTT, 3-(4,5-dimethylthiazol-2-yl)-2,5-diphenyltetrazolium bromide, NQO1, NAD(P)H:quinone oxidoreductase 1, Nrf2/NFE2L2, NF-E2 p45-related factor 2, ROS, reactive oxygen species, SB, silybin, SC, silychristin, SD, silydianin, SFN, sulforaphane, *Silybum marianum*, Silymarin, Flavonolignans, Silybin, Nrf2, NQO1

## Abstract

*Silybum marianum* (milk thistle) is a medicinal plant used for the treatment of various liver disorders. This study examined whether the main flavonolignans from *S. marianum* (i.e. silybin, silychristin, silydianin) and their 2,3-dehydro derivatives (i.e. 2,3-dehydrosilybin, 2,3-dehydrosilychristin, 2,3-dehydrosilydianin) activate the Nrf2 pathway, which regulates the expression of genes encoding many cytoprotective enzymes, including NAD(P)H:quinone oxidoreductase 1 (NQO1). After 48 h of exposure, 2,3-dehydrosilydianin at concentrations of 25 μM and higher significantly elevated the activity of NQO1 in murine hepatoma Hepa1c1c7 cells. In contrast, other tested compounds at non-cytotoxic concentrations had a mild or negligible effect on the NQO1 activity. Using a luciferase reporter assay, 2,3-dehydrosilydianin was found to significantly activate transcription via the antioxidant response element in stably transfected human AREc32 reporter cells. Moreover, 2,3-dehydrosilydianin caused the accumulation of Nrf2 and significantly induced the expression of the *Nqo1* gene at both the mRNA and protein levels in Hepa1c1c7 cells. We found that 2,3-dehydrosilydianin also increased to some extent the expression of other Nrf2 target genes, namely of the heme oxygenase-1 gene (*Hmox1*) and the glutamate-cysteine ligase modifier subunit gene (*Gclm*). We conclude that 2,3-dehydrosilydianin activates Nrf2 and induces Nrf2-mediated gene expression in Hepa1c1c7 cells.

## Graphical abstract

**Figure:**
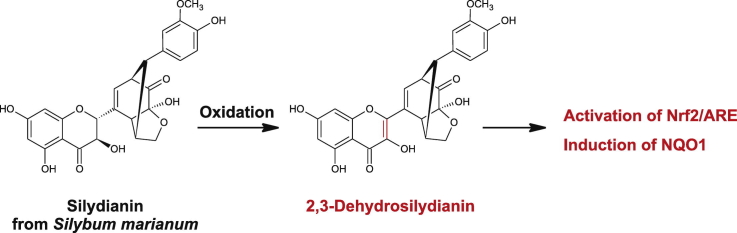

## 1 Introduction

**Fig. 1:**
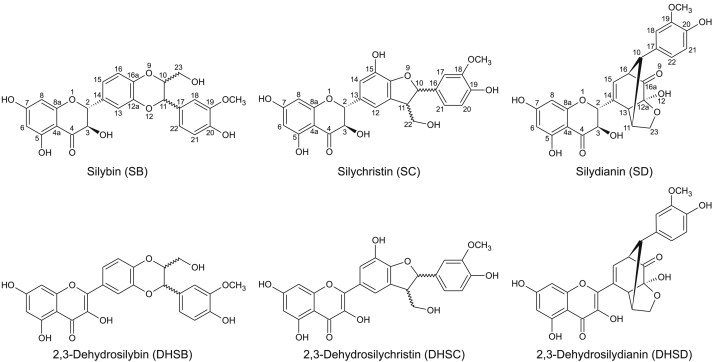
Chemical structures of tested flavonolignans.

Flavonolignans are plant polyphenols with a chemical structure consisting of a flavonoid and a lignan (phenylpropanoid) moiety. They are found in some species of the families Asteraceae, Fabaceae, Poaceae, and others [1]. The best known and most studied flavonolignan is silybin [2], which together with silychristin and silydianin (Fig. 1) are major components of silymarin, a standardized extract from the fruits of *Silybum marianum* (milk thistle; Asteraceae). These flavonolignans originate biosynthetically from the flavanonol taxifolin (2,3-dihydroquercetin) and coniferyl alcohol. Their oxidation at the flavonoid moiety yields the corresponding 2,3-dehydroflavonolignans (Fig. 1), formally derived from the flavonol quercetin. Due to the low stereoselectivity of the biosynthetic processes, silybin, silychristin and also their 2,3-dehydro derivatives naturally occur as pairs of trans-configured diastereomers/enantiomers, denoted A and B [3].

Silymarin is clinically used for its hepatoprotective effects in the complementary therapy of liver disorders caused by various hepatotoxic compounds and viral infections. In addition, anticancer, cardioprotective, neuroprotective, UV-protective, hypocholesterolemic and some other effects have been reported for silymarin in animal models [4], [5], [6], [7]. Although a wide range of molecular targets have been identified in vitro for individual flavonolignans, the protective potential of silymarin is primarily attributed to its antioxidant action [4], [7], [8]. Phenolic compounds exert their antioxidant effects through various mechanisms including *i*) direct scavenging of reactive oxygen species (ROS), *ii*) chelation of transition metal ions, *iii*) inhibition of ROS-generating enzymes, and *iv*) upregulation of antioxidant enzymes [9]. The direct antioxidant activity of silymarin flavonolignans has been investigated repeatedly [10], [11], [12]. In general, silychristin and silydianin appear to be better radical scavengers than silybin [10], [11], and interestingly, 2,3-dehydroflavonolignans exhibit more potent free radical scavenging effects than the corresponding compounds lacking the 2,3-double bond [11], [12]. Silybin has also been characterized as an iron chelator [13] and an inhibitor of prooxidant enzymes such as xanthine oxidase and phagocyte NADPH oxidase [14]. Existing research suggests that the beneficial effects of silymarin could also be associated with the activation of the transcription factor NF-E2 p45-related factor 2 (Nrf2; also called NFE2L2) [15]. Nrf2 controls the antioxidant response element (ARE)-mediated expression of genes encoding various antioxidant and detoxication enzymes such as NAD(P)H:quinone oxidoreductase 1 (NQO1), heme oxygenase-1 (HMOX1), and glutamate-cysteine ligase, composed of a catalytic (GCLC) and modifier (GCLM) subunit [16]. It has been reported that silymarin induces the expression of the *HMOX1* gene in human hepatoma Huh-7 cells [15], and modulates the levels of Nrf2-regulated proteins in animals exposed to various toxic agents [8]. Moreover, the daily oral administration of silybin to Sencar mice for 3–15 days has been shown to elevate the activity of NQO1 in various tissues [17], although the potential involvement of Nrf2 was not investigated. In this study, we examined whether silybin, silychristin, silydianin and their 2,3-dehydro derivatives activate the Nrf2 pathway in cells.

## 2 Materials and methods

### 2.1 Reagents for biological testing

Silybin (SB) was isolated from silymarin (Liaoning Senrong Pharmaceutical, Panjin, China, batch No. 120501) as described previously [18]. Silychristin (SC) and silydianin (SD) were then isolated from the silymarin devoid of SB as described in [18]. 2,3-Dehydrosilybin (DHSB), 2,3-dehydrosilychristin (DHSC) and 2,3-dehydrosilydianin (DHSD) were prepared by the oxidation of SB, SC and SD, respectively. For the preparation of DHSB, see Ref. [19]; for the preparation of DHSC and DHSD, see Ref. [11]. The purity of the tested flavonolignans was at least 95% (HPLC). Dimethyl sulfoxide (DMSO) and sulforaphane were obtained from Sigma-Aldrich (St. Louis, MO, USA).

### 2.2 Cell cultures and treatments

The murine hepatoma Hepa1c1c7 cell line (#95090613, ECACC, Salisbury, UK) was cultured in Minimum essential medium α (M0894, Sigma) supplemented with 2.2 g/L NaHCO_3_ and 10% heat- and charcoal-treated fetal bovine serum (FBS). The stable human mammary AREc32 reporter cell line [20] was cultured in Dulbecco's modified Eagle's medium (#41966, Gibco, Grand Island, NY, USA) supplemented with 2 mM glutamine and 10% FBS. Cells were maintained at 37 °C in a humidified atmosphere containing 5% CO_2_. For experiments, cells were seeded into multiwell plates and the experiments were performed after 24 h of stabilization in fresh complete culture medium. Cells were treated with the tested compounds (in 0.1% (*v/v*) DMSO) and negative controls were treated with 0.1% (*v/v*) DMSO alone.

### 2.3 Cell viability assay

Hepa1c1c7 cells (1 × 10^4^ cells/well in 96-well plates) were treated for 48 h with 0.1% (*v/v*) DMSO (control), 1.5% (*v/v*) Triton X-100 (positive control) or with the tested flavonolignans. After treatment, the cell viability was determined by MTT reduction assay. Cells were washed with phosphate-buffered saline (PBS) and incubated for 2 h at 37 °C in serum-free medium containing 0.5 mg/mL 3-(4,5-dimethylthiazol-2-yl)-2,5-diphenyltetrazolium bromide (MTT). After incubation, the medium was removed and formazan produced by active mitochondria was dissolved in DMSO containing 1% (*v/v*) ammonia. The absorbance at 540 nm was measured on a spectrophotometric plate reader and used to calculate relative cell viability, where cells treated with DMSO alone represented 100% viability.

### 2.4 NQO1 activity assay

After the treatment of Hepa1c1c7 cells (1 × 10^4^ cells/well in 96-well plates), the activity of NQO1 was determined spectrophotometrically as described previously [21]. Cells were washed four times with PBS and lysed with 75 μL of digitonin solution (0.8 g/L digitonin, 2 mM EDTA, pH 7.8) by shaking on an orbital shaker for 20 min at room temperature. One part of the cell lysate (20 μL) was used to determine the protein content. The remaining lysate (55 μL) was mixed with 200 μL of 0.5 M Tris-Cl buffer containing 10% (*w/v*) bovine serum albumin, 1.5% (*v/v*) Tween-20, 7.5 mM FAD, 150 mM glucose-6-phosphate, 2 U/mL glucose-6-phosphate dehydrogenase (Roche Diagnostics, Mannheim, Germany), 50 mM NADP^+^, 25 mM menadione and 0.7 mM MTT. The mixture was incubated for 5 min at room temperature and the reaction was stopped with 50 μL of dicumarol suspension (0.3 mM dicumarol, 5 mM potassium phosphate, 0.5% DMSO). The absorbance of the reduced MTT corresponding to the activity of NQO1 was measured at 610 nm on a spectrophotometric plate reader. The absorbance values were normalized to the protein content and used for the calculation of fold changes versus the control.

### 2.5 Gene reporter assay

AREc32 cells (1 × 10^4^ cells/well in 96-well plates) were treated for 24 h with the tested compounds. After treatment, the plate was frozen and kept at −20 °C for 24 h and then the luciferase activity was measured on a GloMax-Multi+ microplate luminometer (Promega, Madison, WI, USA) using the Bright-Glo Luciferase Assay System (Promega). The luminescence values were normalized to the protein content of the cells and used for the calculation of fold changes versus the control.

### 2.6 Reverse transcription and quantitative real-time PCR

After the treatment of Hepa1c1c7 cells (4 × 10^5^ cells/well in 6-well plates), total RNA was extracted using TRI Reagent Solution (Applied Biosystems, Foster City, CA, USA). RNA samples (2 μg) were reverse-transcribed using a High-Capacity cDNA Reverse Transcription Kit (Applied Biosystems) and real-time PCR was performed in a LightCycler 480 II system (Roche) using TaqMan Universal PCR Master Mix and TaqMan Gene Expression Assays, consisting of specific primers and FAM dye-labeled TaqMan minor groove binder probes (Applied Biosystems). The assay ID was Mm00477784_m1 for Nfe2l2 (Nrf2), Mm01253561_m1 for Nqo1, Mm00516005_m1 for Hmox1, Mm00802655_m1 for Gclc, Mm00514996_m1 for Gclm and Mm99999915_g1 for Gapdh. Amplification conditions were 50 °C for 2 min, 95 °C for 10 min, followed by 40 cycles at 95 °C for 15 s and 60 °C for 1 min. Crossing point values, equivalent to *C*_T_, were determined using second derivative maximum analysis. Relative changes in gene expression were calculated by the comparative *C*_T_ method using the 2^−*ΔΔC*_T_^ equation with results normalized to Gapdh mRNA levels.

### 2.7 Western blot analysis

After the treatment of Hepa1c1c7 cells (4 × 10^5^ cells/well in 6-well plates), total cellular extracts were prepared as described previously [22]. Aliquots containing an equal amount of protein were subjected to sodium dodecyl sulfate-polyacrylamide gel electrophoresis using 4–12% NuPAGE Bis-Tris mini gels (Thermo Fisher Scientific, Waltham, MA, USA), proteins were transferred to polyvinylidene difluoride membrane by electroblotting, and the membranes were probed with appropriate primary antibodies. Rabbit monoclonal Nrf2 (D1Z9C) XP (#12721) antibody was obtained from Cell Signaling Technology (Danvers, MA, USA). Rabbit polyclonal heme oxygenase-1 (sc-10789) and goat polyclonal actin (sc-1616) antibodies were obtained from Santa Cruz Biotechnology (Santa Cruz, CA, USA). Antibodies against NQO1, GCLC and GCLM were kindly provided by Professor John D. Hayes (University of Dundee, Dundee, UK). Primary antibodies were visualized with rabbit anti-goat or goat anti-rabbit horseradish peroxidase-conjugated secondary antibodies using a chemiluminescent reaction. The relative band intensities were determined by densitometric analysis using ImageJ software (National Institutes of Health, Bethesda, MD, USA).

### 2.8 Statistical analysis

Results were expressed as means ± standard deviation. The differences in mean values were analyzed by one-way ANOVA with Tukey post hoc test. A *p* value of < 0.05 was considered to be statistically significant.

## 3 Results and discussion

### 3.1 Effect of tested flavonolignans on NQO1 activity in Hepa1c1c7 cells

**Fig. 2:**
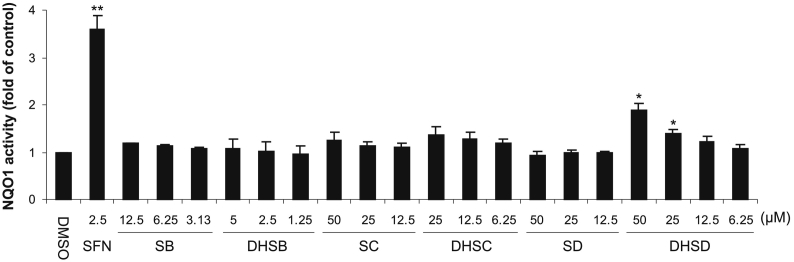
Effect of tested flavonolignans on NQO1 activity in Hepa1c1c7 cells. Cells were treated for 48 h with 0.1% DMSO (control), 2.5 μM sulforaphane (SFN; positive control) or with indicated concentrations of silybin (SB), 2,3-dehydrosilybin (DHSB), silychristin (SC), 2,3-dehydrosilychristin (DHSC), silydianin (SD) or 2,3-dehydrosilydianin (DHSD). After treatment, the activity of NQO1 was determined using the NQO1 assay. Data are means ± standard deviation of three experiments. *p < 0.05; **p < 0.01, significantly increased versus control.

This study was designed to investigate the ability of six flavonolignans to activate the Nrf2 pathway in cells. The study included *i*) silybin, silychristin and silydianin, isolated from commercially available extract from the fruits of *S. marianum* [18], [23], and *ii*) 2,3-dehydrosilybin, 2,3-dehydrosilychristin and 2,3-dehydrosilydianin, which were prepared by oxidation, by molecular oxygen under basic conditions, of the compounds listed in the first item [11], [23]. Flavonolignans could be tested at concentrations of up to 50 μM with respect to their solubility in the cell culture medium, nonetheless the maximum final concentrations of silybin, 2,3-dehydrosilybin and 2,3-dehydrosilychristin were 12.5, 5 and 25 μM, respectively, to avoid cytotoxic effects. To identify whether any of the tested compounds stimulate Nrf2 activity, we evaluated their effect on the activity level of NQO1 in murine hepatoma Hepa1c1c7 cells, a well-established model for the NQO1 assay [21]. NQO1 is a highly-inducible enzyme responsible, among others, for a single-step two-electron reduction of quinones and quinone imines, thus preventing the formation of reactive and toxic semiquinone intermediates [24]. After the exposure of Hepa1c1c7 cells for 48 h to 2.5 μM sulforaphane, a classical Nrf2 activator [25], the activity of NQO1 increased 3.6-fold compared to the control (Fig. 2). Among the tested flavonolignans, only 2,3-dehydrosilydianin was found to produce a significant dose-dependent elevation in NQO1 activity. After 48 h, 25 and 50 μM 2,3-dehydrosilydianin increased the activity of NQO1 to 1.4-fold and 1.9-fold, respectively, compared to the control (Fig. 2). Other tested compounds had mild or negligible effects on the NQO1 activity, even at the highest concentrations tested. After 48 h of incubation, the activity of NQO1 was 1.2-fold with 12.5 μM silybin, 1.1-fold with 5 μM 2,3-dehydrosilybin, 1.2-fold with 50 μM silychristin, 1.3-fold with 25 μM 2,3-dehydrosilychristin, and 0.9-fold with 50 μM silydianin (Fig. 2). Table 1 shows the effect of flavonolignans on the viability of Hepa1c1c7 cells at the maximum concentrations used and after 48 h of exposure. The above results suggest that 2,3-dehydrosilydianin acts as an NQO1 inducer, and thus may potentially activate Nrf2 at non-cytotoxic concentrations.

**Table 1:** Effect of tested flavonolignans on the viability of Hepa1c1c7 cells.

Compound	Concentration	Viability (% of control)
DMSO	0.1%	100
SB	12.5 μM	107.9 ± 9.9
DHSB	5 μM	104.5 ± 10.9
SC	50 μM	122.4 ± 7.7
DHSC	25 μM	96.5 ± 9.0
SD	50 μM	118.3 ± 9.5
DHSD	50 μM	101.5 ± 15.7
Triton X-100	1.5%	0

Hepa1c1c7 cells were treated for 48 h with 0.1% DMSO (control), 1.5% Triton X-100 (positive control) or with indicated concentrations of silybin (SB), 2,3-dehydrosilybin (DHSB), silychristin (SC), 2,3-dehydrosilychristin (DHSC), silydianin (SD) or 2,3-dehydrosilydianin (DHSD). Cell viability was determined by MTT assay. Data are means ± standard deviation of three independent experiments. The table shows the results for the highest flavonolignan concentrations tested in the NQO1 assay.

### 3.2 Activation of Nrf2 by 2,3-dehydrosilydianin in AREc32 reporter cells

**Fig. 3:**
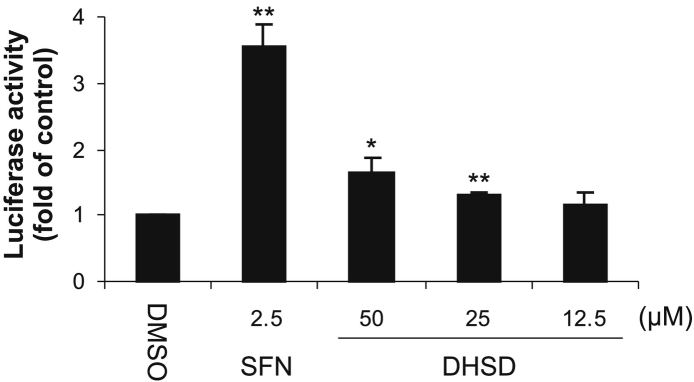
Effect of 2,3-dehydrosilydianin (DHSD) on ARE-driven gene expression in AREc32 reporter cells. Cells were treated for 24 h with 0.1% DMSO (control), 2.5 μM sulforaphane (SFN; positive control) or with 12.5–50 μM DHSD. After treatment, luciferase reporter activity was determined luminometrically and normalized to protein content. Data are means ± standard deviation of three experiments. *p < 0.05; **p < 0.01, significantly increased versus control.

The effect of 2,3-dehydrosilydianin on the transcriptional activity of Nrf2 was examined using stably transfected human AREc32 reporter cells, which contain a luciferase reporter gene controlled by eight copies of the ARE [20]. After the treatment of AREc32 cells for 24 h with 2.5 μM sulforaphane, a positive control, the activity of luciferase was increased 3.6-fold compared to the control (Fig. 3). We found that 2,3-dehydrosilydianin also induced a small but significant elevation in the luciferase activity. AREc32 cells treated for 24 h with 25 and 50 μM 2,3-dehydrosilydianin exhibited a 1.3-fold and 1.6-fold increase in the luciferase activity, respectively, compared to the control (Fig. 3). These results confirm that 2,3-dehydrosilydianin can activate Nrf2-dependent transcription in cells.

### 3.3 Accumulation of Nrf2 by 2,3-dehydrosilydianin in Hepa1c1c7 cells

**Fig. 4:**
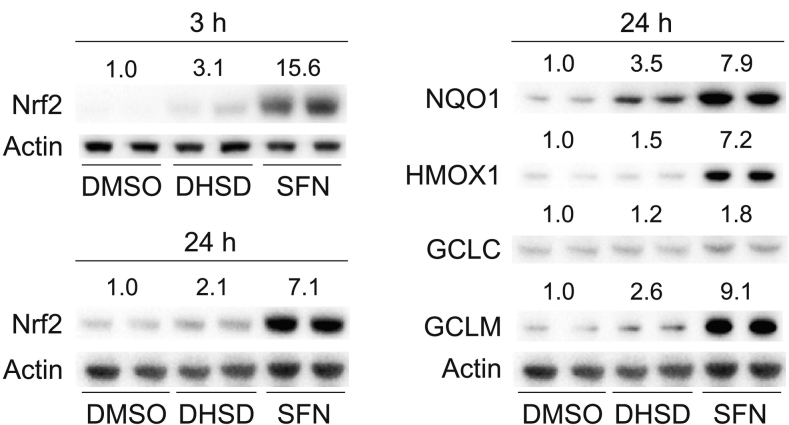
Effect of 2,3-dehydrosilydianin (DHSD) on the levels of Nrf2 and Nrf2-regulated proteins in Hepa1c1c7 cells. Cells were treated for 3 or 24 h (as indicated) with 0.1% DMSO (control), 5 μM sulforaphane (SFN; positive control) or 50 μM DHSD. After treatment, the protein levels of Nrf2, NQO1, HMOX1, GCLC, GCLM, and actin in the whole cell lysates (30 μg/lane) were analyzed in duplicate by Western blotting. Representative Western blots are shown. Relative band intensities were determined densitometrically and normalized to actin. Data expressed as folds of control are means of three experiments.

The activity of Nrf2 depends on the interaction between Nrf2 and its negative regulator, Kelch-like ECH-associated protein 1 (Keap1). Under homeostatic conditions, Keap1 binds to Nrf2 and targets it for ubiquitination and proteasomal degradation. In contrast, stress conditions associated with the oxidation or covalent modification of cysteine residues in Keap1 and/or with the phosphorylation of Nrf2 inactivate Keap1 and thus stabilize Nrf2, which in turn accumulates in the cell and increases the expression of Nrf2 target genes [26]. Using quantitative real-time PCR and Western blot analysis, we found that 50 μM 2,3-dehydrosilydianin as well as 5 μM sulforaphane, a positive control [27], elevated the protein levels of Nrf2 in Hepa1c1c7 cells after 3 and 24 h of exposure (Fig. 4), while the levels of Nrf2 mRNA remained unaffected, as shown after 6 h (Table 2). Although the effect of 2,3-dehydrosilydianin on the level of Nrf2 was much weaker than that of sulforaphane (Fig. 4), the accumulation of Nrf2 corroborates the ability of 2,3-dehydrosilydianin to increase the activity of Nrf2.

**Table 2:** Effect of 2,3-dehydrosilydianin (DHSD) on the expression of Nrf2 and Nrf2 target genes in Hepa1c1c7 cells.

Compound	Concentration	Nrf2 mRNA (fold of control)	Nqo1 mRNA (fold of control)	Hmox1 mRNA (fold of control)	Gclc mRNA (fold of control)	Gclm mRNA (fold of control)
DMSO	0.1%	1	1	1	1	1
SFN	5 μM	0.8 ± 0.1	3.9 ± 0.4⁎⁎⁎	12.4 ± 0.8⁎⁎⁎	2.5 ± 0.4⁎⁎	4.7 ± 1.0⁎⁎
DHSD	25 μM	1.1 ± 0.1	1.6 ± 0.2⁎	1.2 ± 0.1	1.0 ± 0.0	1.0 ± 0.0
DHSD	50 μM	1.1 ± 0.2	2.3 ± 0.5⁎	2.2 ± 0.6	1.3 ± 0.2	1.5 ± 0.3

Hepa1c1c7 cells were treated for 6 h with 0.1% DMSO (control), 5 μM sulforaphane (SFN; positive control) or with 25 and 50 μM DHSD. The levels of Nrf2, Nqo1, Hmox1, Gclc and Gclm mRNA were determined by quantitative real-time PCR with the results normalized to Gapdh mRNA. Data are means ± standard deviation of three experiments.

⁎ *p* < 0.05.

⁎⁎ *p* < 0.01.

⁎⁎⁎ *p* < 0.001, significantly increased versus control.

### 3.4 Effect of 2,3-dehydrosilydianin on the expression of Nrf2 target genes in Hepa1c1c7 cells

To further investigate the effect of 2,3-dehydrosilydianin on Hepa1c1c7 cells, we analyzed the expression of selected Nrf2 target genes, including *Nqo1*, *Hmox1*, *Gclc* and *Gclm*. As expected, 5 μM sulforaphane (a positive control) [27] significantly increased the expression of all four genes after 6 h of exposure (Table 2), and also upregulated the protein levels of NQO1, HMOX1, GCLC and GCLM after 24 h (Fig. 4). In Hepa1c1c7 cells treated for 6 h with 2,3-dehydrosilydianin, we found a significant increase in the expression of the *Nqo1* gene. At concentrations of 25 and 50 μM, 2,3-dehydrosilydianin elevated Nqo1 mRNA levels to 1.6-fold and 2.3-fold, respectively, compared to the control. The expression of the other tested genes was also affected to some extent by 2,3-dehydrosilydianin, but only at a concentration of 50 μM, where the increase in mRNA levels of Hmox1, Gclc and Gclm were 2.2-fold, 1.3-fold and 1.5-fold, respectively (Table 2). Western blot analysis showed that the changes in gene expression induced in Hepa1c1c7 cells by 50 μM 2,3-dehydrosilydianin were accompanied by an obvious increase in the protein levels of NQO1 and GCLM, while the levels of HMOX1 and GCLC remained almost unchanged after 24 h of exposure (Fig. 4). These results show that 2,3-dehydrosilydianin activates Nrf2-dependent gene expression. However, this effect is clearly evident only in the induction of a highly inducible *Nqo1* gene [28], and thus 2,3-dehydrosilydianin is a considerably weaker Nrf2 activator than sulforaphane, which served as a positive control in the study. Although our results cannot explain the lower potency of 2,3-dehydrosilydianin compared to sulforaphane, we may presume that the two compounds differ in the mechanism by which they activate Nrf2. The Nrf2 activation by sulforaphane, a natural isothiocyanate, results from the covalent modification of cysteine residues of Keap1 by direct reaction with the electrophilic isothiocyanate group [26]. On the other hand, the effect of 2,3-dehydrosilydianin could be mediated through a more complex mechanism dependent on the phosphorylation of Nrf2 by some protein kinase. This kind of mechanism is involved in the Nrf2 activation by structurally related compounds such as quercetin [29] and quercetin-7-gallate [27].

## 4 Conclusions

Our study examined the effect of six flavonolignans, namely of silybin, silychristin, silydianin and of their 2,3-dehydro derivatives, on the Nrf2 pathway. We have demonstrated that 2,3-dehydrosilydianin, in contrast to the other tested compounds, activates the Nrf2-dependent gene expression in vitro. This is mainly documented by the activation of the antioxidant response element in AREc32 reporter cells, and by the accumulation of Nrf2 and induction of NQO1 at the mRNA, protein and activity levels in Hepa1c1c7 cells. The difference in the effect of 2,3-dehydrosilydianin and silydianin confirms the importance of the 2,3-double bond in the structure of flavonolignans for certain biological activities. For instance, 2,3-dehydrosilydianin was shown to inhibit *tert*-butyl hydroperoxide-induced lipid peroxidation with a higher potency than silydianin [11]. Similarly, 2,3-dehydrosilybin is a more potent inhibitor of DNA topoisomerase I [30], cytochrome P450 1A1 [31] and the glucose transporter GLUT4 [32] than silybin.

Since the main silymarin components silybin, silychristin and silydianin did not activate the Nrf2 pathway, our results cannot explain the effects of silymarin and silybin on the expression and/or activity of Nrf2-regulated proteins observed in vivo [8], [17]. We can, however, speculate that some effects of silymarin may be mediated by minor silymarin flavonolignans (e.g. isosilybin) or by non-flavonolignan components [3], [4], of which the flavonoids taxifolin and quercetin were recognized as Nrf2 activators [27], [33], [34]. Moreover, the formation of potentially active metabolites should also be taken into account when interpreting the in vivo bioactivity of silymarin and flavonolignans. Accordingly, we suggest that further research on 2,3-dehydrosilydianin should be aimed at evaluating its effect on the Nrf2 pathway in vivo and identifying its metabolites.

## Conflict of interest

The authors declare that there are no conflicts of interest.
